# Body Surface Area and Baseline Blood Pressure Predict Subclinical Anthracycline Cardiotoxicity in Women Treated for Early Breast Cancer

**DOI:** 10.1371/journal.pone.0165262

**Published:** 2016-12-02

**Authors:** Paul Kotwinski, Gillian Smith, Jackie Cooper, Julie Sanders, Louise Ma, Albert Teis, David Kotwinski, Michael Mythen, Dudley J. Pennell, Alison Jones, Hugh Montgomery, on behalf of the Breast cancer Early disease: Toxicity from Therapy with Epirubicin Regimens–Cardiac Assessment and Risk Evaluation (BETTER-CARE) Study Investigators

**Affiliations:** 1 Department of Cardiology, North and East Hertfordshire NHS Trust, Stevenage, United Kingdom; 2 Institute for Human Health and Performance, University College London, London, United Kingdom; 3 NIHR Cardiovascular Biomedical Research Unit, Royal Brompton Hospital and Imperial College, London, United Kingdom; 4 Centre for Cardiovascular Genetics, University College London, London, United Kingdom; 5 Barts Heart Centre, St Bartholemew’s Hospital, Barts Health NHS Trust, London, United Kingdom; 6 Cardiovascular Imaging Unit, Cardiology Department, Germans Trias i Pujol University Hospital, Badalona. Barcelona, Spain; 7 The Anaesthetic Department, Derriford Hospital, Plymouth, United Kingdom; 8 Department of Anaesthesia and Critical Care, University College London Hospital, London, United Kingdom; 9 LOC, London, United Kingdom; 10 UCL Institute of Cardiovascular Science and NIHR University College London Hospitals Biomedical Research Centre, London, United Kingdom; University of Miami School of Medicine, UNITED STATES

## Abstract

**Background and Aims:**

Anthracyclines are highly effective chemotherapeutic agents which may cause long-term cardiac damage (chronic anthracycline cardiotoxicity) and heart failure. The pathogenesis of anthracycline cardiotoxicity remains incompletely understood and individual susceptibility difficult to predict. We sought clinical features which might contribute to improved risk assessment.

**Methods:**

Subjects were women with early breast cancer, free of pre-existing cardiac disease. Left ventricular ejection fraction was measured using cardiovascular magnetic resonance before and >12 months after anthracycline-based chemotherapy (>3 months post-Trastuzumab). Variables associated with subclinical cardiotoxicity (defined as a fall in left ventricular ejection fraction of ≥5%) were identified by logistic regression.

**Results:**

One hundred and sixty-five women (mean age 48.3 years at enrollment) completed the study 21.7 months [IQR 18.0–26.8] after starting chemotherapy. All received anthracyclines (98.8% epirubicin, cumulative dose 400 [300–450] mg/m^2^); 18% Trastuzumab. Baseline blood pressure was elevated (≥140/90mmHg, mean 147.3/86.1mmHg) in 18 subjects. Thirty-four subjects (20.7%) were identified with subclinical cardiotoxicity, independent predictors of which were the number of anthracycline cycles (odds ratio, OR 1.64 [1.17–2.30] per cycle), blood pressure ≥140/90mmHg (OR 5.36 [1.73–17.61]), body surface area (OR 2.08 [1.36–3.20] per standard deviation (0.16m^2^) increase), and Trastuzumab therapy (OR 3.35 [1.18–9.51]). The resultant predictive-model had an area under the receiver operating characteristics curve of 0.78 [0.70–0.86].

**Conclusions:**

We found subclinical cardiotoxicity to be common even within this low risk cohort. Risk of cardiotoxicity was associated with modestly elevated baseline blood pressure–indicating that close attention should be paid to blood pressure in patients considered for anthracycline based chemotherapy. The association with higher body surface area suggests that indexing of anthracycline doses to surface area may not be appropriate for all, and points to the need for additional research in this area.

## Introduction

Anthracyclines remain the mainstay of systemic chemotherapy for many malignancies including breast cancer [1]. Whilst clinically effective, such therapy can cause irreversible cardiac injury (type I cardiotoxicity) resulting in ‘*chronic progressive anthracycline cardiotoxicity’* (*cAC*) and thence premature heart failure, the prevalence of which rises with the time following treatment [1, 2]. Though *population* risk of cardiotoxicity rises with cumulative dose and the prevalence of cardiovascular risk factors, *individual* susceptibility is highly idiosyncratic, incompletely understood and difficult to predict [3, 4]. Furthermore, current tests are inadequate for risk stratification: serial measurement of LV ejection fraction (LVEF) only identifies cardiotoxicity after significant damage has been incurred [5], while the use of biomarkers remains to be validated [6, 7]. As a result, anthracyclines continue to cause heart failure in some (at perceived low risk), whilst their use is restricted in others who might benefit [4]. A more complete understanding of the factors underlying susceptibility to *cAC*, and (ultimately) the construction of predictive models, might help guide management by influencing choice of treatment regimen, targeting prophylaxis, or selecting individuals for cardiac surveillance during chemotherapy and in the longer term [3, 8]. We thus sought mechanistic insights into the pathogenesis of *cAC* and to define elements which might contribute to increased risk, using these to construct a predictive model.

## Materials and Methods

The study had ethics approval from the South East England Multi-Regional Ethics Committee. Informed, written consent was obtained from all participants.

### Participants

Recruitment was from 12 centres (see Acknowledgments) through the UK’s National Cancer Research Network (NCRN). This research forms part of a *prospective gene-environment interaction* study seeking the association of gene variants with cardiotoxicity (results of which will soon be submitted for publication). The power of such studies relies on cohort homogeneity, which amplifies the relative effect of remaining variables (genetic and non-genetic) [9]. Susceptibility to *cAC* is influenced by gender, race, age, cardiovascular disease and risk factors, cardiac medications, and anticancer regimen [1, 10]. Entry criteria (Table 1) balanced the desired homogeneity against feasibility of recruitment. Eligible were anthracycline naïve women aged >18 years without pre-existing cardiac disease, and with planned anthracycline chemotherapy for early breast cancer. Excluded were those of non-European ethnicity, or with potentially confounding comorbidities such as diagnosed hypertension, diabetes, BMI ≥35 kg/m^2^ and renal impairment. For practical and ethical reasons, eligible women attending for cardiovascular magnetic resonance (CMR) continued in the study, even were confounding factors later recognised. Treatment regimens were determined by the attending clinicians at the recruiting centres, uninfluenced by study participation.

**Table 1:** Eligibility Criteria.

**Inclusion Criteria**
• Female gender
• Age ≥18 years
• White/European ethnicity
• Histologically-proven, early breast cancer
• Planned adjuvant or neoadjuvant anthracycline-based chemotherapy
**Exclusion Criteria**
• Contraindications to cardiovascular magnetic resonance
• Pre-existing cardiac disease^†^ including: heart failure, cardiomyopathy, coronary disease, audible murmur, valvular disease, arrhythmias, pacemaker or defibrillator.
• Previous anthracycline chemotherapy
• Bilateral breast surgery (difficult venous cannulation for CMR)
• Anticipated high dose-volume cardiac irradiation, or internal mammary node irradiation
• Diagnosed hypertension or booking blood pressure ≥160/100^‡^ mmHg
• Diabetes mellitus
• Cerebrovascular disease
• Peripheral vascular disease
• Body mass index (BMI) ≥35^‡^ kg/m^2^
• History of pulmonary embolism
• Serum Creatinine >120μmol/L
• Bilirubin > 17μMol/l, AST or ALT >45 iu/L
• History of intravenous drug abuse or prolonged alcohol abuse
• Known HIV infection
• Uncorrected hypo/hyperthyroidism
• Haemoglobin <100 g/l
• Drugs with cardiovascular effects including ACE inhibitors, beta-blockers, antihypertensive, anti-anginal, anti-arrhythmic and diuretic agents

^†^Including significant abnormalities identified on baseline CMR.

^‡^Selecting cut-offs of BMI ≥35 as kg/m^2^ and a booking blood pressure measurement ≥160/100^‡^ mmHg reflected the need to balance desired cohort homogeneity against feasibility of recruitment

### Study Size and Timelines

Subjects were recruited between June 2005 and May 2009. A target of 276 subjects was based upon the requirements for study scale of the funding body and experience-informed power estimates for genetic study [9, 11]. Recruitment was slower than anticipated, and the study thus closed with 196 patients enrolled.

### Cardiovascular Magnetic Resonance (CMR)

CMR was undertaken pre- and ≥12 months post-anthracycline chemotherapy (≥3 months after completing Trastuzumab). Scans were performed on 1.5 Tesla scanners (Siemens Medical Systems, Erlangen, Germany) at 3 centres (see acknowledgments). Cine images were acquired, for assessment of left ventricular ejection fraction and mass, using a steady state free precession sequence with retrospective ECG gating [12]. Late gadolinium-enhanced images were obtained at baseline and routinely at follow-up until October 2008 (following interim review of results). Gadolinium diethylenetriamine penta-acetic acid (Gd-DPTA, Magnevist, Schering) was administered as a 0.1mmol/Kg bolus dose via a peripheral cannula. Contrast-enhanced images were acquired 10 minutes post-injection using an *inversion-recovery segmented gradient echo* (Turbo-Flash) sequence[13]. Analysis of paired CMR scans (images side-by-side) was performed by a single investigator (PK, who was blind to identity, clinical, temporal and research data) using semi-automated PC-based software (CMRtools Cardiovascular Imaging Solutions, London, UK) [12, 13]

The primary outcome measure was change in left ventricular ejection fraction (LVEF), an absolute fall ≥5% defining (*a priori*) the ‘*subclinical cardiotoxicity’* group. All other participants comprised the ‘*minimally/unaffected control’* group. We defined *overt anthracycline cardiotoxicity* as the diagnosis of heart-failure by the clinical team, or a subclinical fall in LVEF ≥10% to below normal using age-gender-CMR specific reference ranges [12]. This being more appropriate than using 53% (the recommended lower limit of normal using 2D echocardiography[14]) because normal values differ between imaging modalities[15]. This issue is recognised in the 2014 expert consensus report on multimodality imaging in adult cancer patients, which also endorses CMR as the ‘reference standard’ for measuring LVEF[14].

### Clinical Data

**Fig 1:**
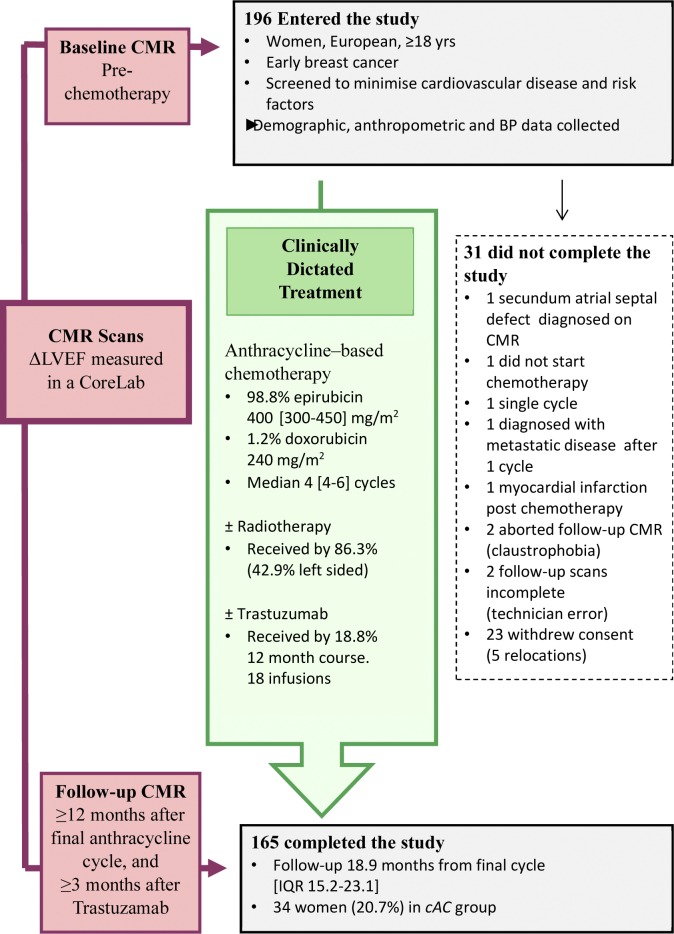
Summary of study design.

The data collection protocol is outlined in Fig 1. Baseline demographic and anthropometric data were recorded. Seated blood pressure was measured using automated sphygmomanometers, after 5 minutes rest. Baseline blood pressure (BP) was the mean of measurements at enrolment (oncology unit, 1 or 2 averaged readings) and CMR (1 reading). Percentage body fat was measured at the CMR Unit (BC531 Fitness Bioimpedence Innerscan Body Composition monitor, Tanita Corporation, Tokyo, Japan); equipment failure meant this was only recorded in a proportion of subjects. Body mass index (BMI: weight in kg/ (height in metres)^2^) and body surface area (BSA by Dubois formula: 0.007184 × (height in cm)^0.725^ × (weight in kg)^0.425^)) were calculated. Estimated glomerular filtration rate (eGFR) was calculated using the modified diet in renal disease (MDRD) formula [16]

### Statistical Analysis

Analysis was undertaken using Stata version 12 (StataCorp Texas). Differences in mean values were assessed using unpaired t-tests or with the Mann-Whitney U test for non-normally distributed data. Categorical data were compared using Chi Squared and Fisher’s exact tests. Best discriminator values were defined by receiver operator curve analysis. Odds ratios and p-values were obtained from logistic regression models; where numbers were small, exact logistic regression was used. To determine independent predictors of cardiotoxicity, variables were selected using stepwise multiple logistic regression with backwards selection, removing terms with p ≥0.1 and adding those with p <0.05. Patients with missing data were excluded. The models were validated with bootstrap re-sampling; all terms retained in the model were selected in at least 65% of the bootstrap samples.

## Results

Of the 196 women enrolled in the study, 165 completed follow-up 21.7 months later [IQR 18.0 to 26.8], as shown in Fig 1. Their mean age at enrolment was 48.3 years. Other baseline characteristics are shown in Table 2. All were in sinus rhythm; none had myocardial infarction, cardiomyopathy or valvular dysfunction evident on CMR. All received anthracycline based chemotherapy (98.8% epirubicin, median cumulative dose 400 [300–450] mg/m^2^) and 18% a sequential 12-month course of Trastuzumab (further details in S1 Table). Following treatment, LVEF fell by (mean ± SEM) 2.1±0.3% in the cohort overall (p<0.0001). There were 34 subjects (20.7%) in the *cAC group*, and 131 (79.3%) to the *minimally/unaffected* control group, with falls in LVEF of 7.7±0.4% (p<0.0001) and 0.6±0.3% (p = 0.02) respectively. No subjects developed *overt anthracycline cardiotoxicity*. No focal fibrosis was identified using late gadolinium enhancement (imaged in 120 at follow-up). Additional CMR data are presented in S2 Table.

**Table 2:** Participant characteristics pre-chemotherapy.

	Mean (SD)	%
Age (years)	48.3 (8.9)	
Age≥60 years		11.5%
European ethnicity^†^		98.2%
Height (m)	1.64 (0.07)	
Weight* (kg)	66.3 (12.1)	
BSA* (m^2^)	1.72 (0.16)	
BSA >2.00 (m^2^)		6.7%
BMI* (kg/m^2^)	24.7 (4.2)	
BMI ≥30 kg/m^2^		14.6%
BMI ≥35 kg/m^2^^†^		3.6%
Systolic BP (mmHg)	117.5 (15.4)	
Diastolic BP (mmHg)	71.7 (10.3)	
Diagnosed hypertension^†^		1.8%
Baseline BP ≥160/100^†^		1.8%
Baseline BP ≥140/90mmHg		11.0%
Diabetes^†^		0.6%
Creatinine (μmol/l)	68.2 (9.8)	
eGFR (ml/min/1.73m^2^)	86.4 (15.4)	
ACE inhibitor, ARB or beta-blocker		0%

Compared with the general population of chemotherapy-treated breast cancer patients, the cohort was young with a low prevalence of cardiovascular risk factors.

*Geometric mean (approximate SD). ARB: angiotensin receptor blocker.

^†^Exclusion criteria identified after attending for CMR. As per protocol, these subjects continued in the study.

Several factors were associated with subclinical *cAC* on univariate analysis (Tables 3 and 4). Women in the *cAC* group were more likely to have received a cumulative epirubicin dose ≥450mg/m^2^ (odds ratio (OR) 2.21 [1.01–4.82], p = 0.047), and a greater number of anthracycline cycles (median 6 vs 4, p = 0.03). The interval between completing anthracycline treatment and follow-up was on average longer (median of 20.7 vs. 18.3 months, p = 0.04), the magnitude of the difference in part being explained by the numerically higher proportion of the *cAC* group receiving Trastuzumab therapy (26.5% versus 16.8%, p = 0.20). However, excluding those who received Trastuzumab, a longer interval to follow-up remained associated with risk of subclinical cardiotoxicity: OR 1.06 [1.00–1.12] per month. Subclinical cardiotoxicity was also associated with increasing weight, height and BSA, the *cAC* group being an average 6kg heavier (p = 0.01) and 4cm taller (p = 0.007), with a 0.1m^2^ higher BSA (p = 0.003). Women with a high BSA (>2.00m^2^) were significantly more likely to experience a fall in LVEF ≥5% (OR 3.59 [1.02–12.58], p = 0.046). BMI and the proportion of obese individuals were not statistically different between groups. Body fat was numerically greater in the *cAC* group, but this failed to reach statistical significance (p = 0.07, data in 118 subjects). Those with a body percentage fat ≥31% were more likely to be in the *cAC* group (cut-off identified by ROC analysis, OR 3.26 (1.20–8.86), p = 0.02). Blood pressure was elevated (≥140/90mmHg) in 18 subjects (11.0%, one diagnosed/ treated for hypertension). Women with elevated BP (mean 147.3/86.1mmHg) were more likely than those with normal BP (<140/90mmHg, mean 113.8/69.9mmHg) to be in the *cAC* group: OR 3.69 [1.33–10.26] (p = 0.01).

**Table 3:** Treatment factors and cardiotoxicity.

Variable	*cAC* group		Control group		Univariate Association	
	Median [IQR]	%	Median [IQR]	%	OR^1^ (95%CI)	P value
Cumulative epirubicin /BSA ≥450mg/m^2^		60.6%		41.1%	2.21 (1.01–4.82)	0.047
Cumulative epirubicin dose / BSA (mg/m^2^)	450 [360–450]		400 [300–450]		1.12 (0.72–1.75)	0.61
Number anthracycline cycles	6 [4–6]		4 [4–6]		1.36 (1.01–1.84)	0.03
Radiotherapy		78.1%		88.4%	0.47 (0.17–1.27)	0.14
Left-sided radiotherapy		38.2%		44.2%	0.78 (0.36–1.70)	0.53
Trastuzumab		26.5%		16.8%	1.78 (0.73–4.34)	0.20
Aromatase inhibitor		14.7%		13.0%	1.08 (0.63–1.84)	0.79
Tamoxifen		41.2%		55.0%	0.57(0.27–1.23)	0.15
Interval from final anthracycline cycle to follow-up (months)	20.7 [17.3–27.3]		18.3 [14.9–22.1]		1.05 (1.00–1.09)	0.04
Interval from final anthracycline cycle–Trastuzumab untreated subjects only (months)	18.1 [17.1–27.0]		17.1 [14.6–20.9]		1.06 (1.00–1.12)	0.04

^1^OR for a 100mg/m^2^ dose increase and 1 month longer follow-up.

**Table 4:** Patient factors and cardiotoxicity.

Variable	*cAC* group		Control group		Univariate association	
	Mean (SD)	%	Mean (SD)	%	OR (95% CI)	P value
Age	48.7 (7.6)		48.2 (9.2)		1.06 (0.72–1.55)*	0.77
Age≥60 years		2.9%		13.7%	0.19 (0.00–1.31)	0.13
Height (m)	1.67(0.07)		1.63 (0.06)		1.70 (1.14–2.52)*	0.008
Weight^†^ (kg)	71.1 (14.5)		65.1 (11.2)		1.60 (1.12–2.30)*	0.01
BSA (m^2^)^†^	1.80 (0.18)		1.70 (0.15)		1.78 (1.21–2.61)*	0.003
BSA >2.00 (m^2^)		14.7%		4.6%	3.59 (1.02–12.58)	0.046
BMI^†^ (kg/m^2^)	25.6 (4.9)		24.5 (4.0)		1.31 (0.90–1.90)*	0.16
BMI≥30 kg/m^2^		20.6%		13.0%	1.74 (0.66–4.61)	0.27
Body Fat (%)^‡^	34.8 (7.1)		31.7 (7.6)		1.51 (0.97–2.36)*	0.07
Body Fat ≥ 31%^¶^		76.9%		50.0%	3.26 (1.20–8.86)	0.02
eGFR (ml/min/1.73m^2^)	82.6 (14.0)		87.5 (15.6)		0.72 (0.48–1.06)*	0.10
Diabetes		0.0%		0.8%		1.00
Diagnosed Hypertension		2.9%		1.5%		0.50
Systolic BP (mmHg)	121.4(17.8)		116.5 (14.6)		1.36 (0.94–1.97)*	0.10
Diastolic BP (mmHg)	73.7 (13.1)		71.2 (9.4)		1.27 (0.87–1.86)*	0.21
BP ≥140/90mmHg		23.5%		7.7%	3.69 (1.33–10.26)	0.01
BP <100/60mmHg		8.8%		18.5%	0.43 (0.08–1.56)	0.27

*Odds ratios are for a 1 SD increase.

†Data log transformed, geometric means (approximate SDs). Blood pressure (BP) measured in 164 women (163 at enrolment, 143 with CMR)

‡Measurements available for 118 subjects

¶Best discriminator value defined by receiver operator curve (ROC) analysis.

On multivariate analysis (Table 5), the independent predictors of cardiotoxicity were the number of anthracycline cycles (OR 1.64[1.17–2.30] per additional cycle), higher BSA (OR 2.08 [1.36–3.20] per standard deviation (0.16m^2^) increase), elevated BP (OR 5.36 [1.73–17.61]) and Trastuzumab therapy (OR 3.35 [1.18–9.51]). The resultant model had an area under the receiver operating characteristics (ROC) curve of 0.78 (0.70–0.86).

**Table 5:** Multivariate logistic regression modeling.

Variable	OR (95% CI)	P value
Number of anthracycline cycles	1.64 (1.17–2.30) ^†^	0.004
BSA (m^2^)	2.08 (1.36–3.20) ^‡^	0.001
Elevated BP (≥140/90mmHg)	5.36 (1.73–17.61)	0.006
Trastuzumab therapy	3.35 (1.18–9.51)	0.02
**Model performance indices**		
Area under ROC curve	0.78 (0.70–0.86)	
Akaike Information Criterion (AIC)	145.0	
Positive predictive value, PPV (<5% FPR)	62.5%	
Negative predictive value, NPV (<5% FPR)	84.1%	

FPR, False positive rate.

†OR per cycle.

‡OR per standard deviation (0.16 m2) increase in body surface area (BSA). Analysis undertaken using the 161 subjects who received epirubicin and had complete dose and blood pressure (BP) data, of whom 33 were in the cAC group. The input variables were: epirubicin ≥450mg/m2 (yes/no), number of anthracycline cycles, height, weight, BSA (as continuous variable and >2.00), BP ≥140/90mmHg (yes/no) and BP<100/60mmHg (yes/no), Trastuzumab (yes/no), and Interval from final anthracycline cycle. For calculation of model score: yes = 1 and no = 0. Model 1 score = 0.4955 x (number of cycles) +4.552 x (body surface area) +1.679 x (blood pressure≥140/90) +1.209*(Trastuzumab). The same variables were retained in the model if baseline LVEF was used as an input, thus confirming minimal confounding effect of regression towards the mean. A 2nd analysis performed to exclude any influence of the time to follow-up (by omitting both the interval and Trastuzumab treatment status) performed similarly: area under the ROC curve of 0.74 (0.65–0.83), positive predictive value (PPV) 64.7%, negative predictive value (NPV) 84.7%.

## Discussion

We recruited a highly selected cohort of patients, who were young and with a low prevalence of comorbidity and cardiovascular risk factors relative to the wider breast cancer population. Using CMR, we found that 1 in 5 of the cohort experienced a fall in LVEF of at least 5% following anthracycline chemotherapy, despite being at low risk for cardiotoxicity. Confirming data from others, cardiotoxicity risk related to cumulative anthracycline dose, Trastuzumab therapy and elevated blood pressure. We have also shown, for the first time, that risk also rises with increasing BSA.

### Observed anthracycline cardiotoxicity

CMR is the gold standard for serial measurement of LVEF, being superior to 2-dimensional echocardiography (better inter-study reproducibility allowing a 5% fall in LVEF to be reliably detected, while increasing study power) and radionuclide ventriculography (detection of structural heart disease and avoidance of ionising radiation) [11, 14, 17]. CMR studies have shown that LVEF normally rises by 0.1% per year, because of age-related cardiac remodelling [12, 18]. This relationship was observed in our cohort pre-chemotherapy, LVEF being 0.14% higher per year of age (linear regression, p<0.001). However, following treatment, LVEF deviated from this trajectory, instead *falling* by a mean of 2.1% over an average period of 21.7 months (p<0.0001). The majority of this cardiotoxicity was accounted for by the *cAC group* (mean fall 7.7%), while the ‘*minimally/unaffected’* control group suffered no substantial impact (mean fall 0.6%).

The fact that no patients in our cohort developed overt anthracycline cardiotoxicity likely reflects the very low prevalence of comorbidities in the cohort, their relative youth, and the fact that the epirubicin doses used in early breast cancer are well below the 900mg/m^2^ empiric dose cap.

Of note, it is not standard UK practice to routinely undertake cardiac imaging in patients receiving adjuvant anthracycline chemotherapy. Thus, no such data are available to augment those which we present. The 31 patients who received sequential Trastuzumab did have cardiac imaging (a mixture of non-standardised echocardiography and radionuclide ventriculography) at their different centres before and during Trastuzumab treatment (see below). Scanning during Trastuzumab therapy primarily detects type II (reversible) cardiotoxicity, not the type I cardiotoxicity that was the focus of our study.

### Influence of cumulative anthracycline dose and time from treatment on cardiotoxicity risk

The identification of the characteristic cumulative dose-dependency of *cAC* (OR 2.21 [1.01–4.82] at ≥450mg/m^2^ epirubicin) confirms the discriminative power of the study model. Although retention of *number of anthracycline cycles* on multivariate analysis (rather than cumulative dose) might signal that additional insults and/or the total duration of injury also contribute to *cAC* risk, the relationship was primarily driven by cumulative dose (Spearman correlation, r = 0.90, p<0.001). Evidence for the progressive nature of *cAC* was also observed, the OR for cardiotoxicity in those who did not receive Trastuzumab being 1.06 [1.00–1.12] per month post-treatment. The dataset and the spread of follow-up are, however, insufficient to accurately project the long-term course of decline.

### Influence of anthropometric factors on cardiotoxicity risk

Increasing height, weight and BSA were all associated with increasing *cAC* risk on univariate analysis. On multivariate analysis, BSA was the best discriminator amongst the anthropometric factors when considered as a continuous variable (OR for cardiotoxicity of 2.08 [1.36–3.20] per standard deviation (0.16m^2^) increase). We are the first to report this association, which may be of clinical relevance suggesting as it does that BSA-adjusted dosing of anthracyclines (accepted standard practice) may increase cardiotoxicity risk. Such an effect is biologically plausible, were cardiomyocytes exposed to higher peak and/or cumulative anthracycline doses. Such a possibility has previously been suggested [19], while historically (unproven) concerns about increased risk of toxicity led some oncologists to apply an empirical dose cap at a BSA of 2m^2^ [20]. Our observation that BSA >2.00m^2^ was associated with subclinical cardiotoxicity (OR 3.59 [1.02–12.58] on univariate analysis) is thus noteworthy. No alternative explanation to BSA-indexing is apparent for the observed association between *cAC* risk and increasing height. Meanwhile, higher weight and obesity (which have previously been associated with cardiotoxicity[21, 22] might have more direct influence on cardiotoxicity risk given that body composition (specifically the proportion of lean and adipose tissues) affects the pharmacokinetics/dynamics of anticancer drugs [20, 23, 24]. In addition, non-pharmacokinetic mechanisms might also play a role, given that being overweight or obese are risk factors for developing heart failure [25]. Although BMI was not associated with cardiotoxicity in our study, it is a poor predictor of body composition [26], and it is noteworthy in this regard that those with body fat ≥31% were more likely to be in the *cAC* group (p = 0.02). In addition, the selected nature of our cohort, which included very few women with BMI >35kg/m^2^, may have biased against a signal. Nonetheless, the relationship between body morphology and composition with anthracycline/Trastuzumab cardiotoxicity has become a matter of increasing interest, with a detailed systematic review and meta-analysis being only recently published [27]. In keeping with our findings, this concludes that ‘overweight and obesity are risk factors for cardiotoxicity from anthracyclines and sequential anthracyclines and trastuzumab’. Such associations are postulated to be driven in part by reduced levels of the adipocytokine (adipocyte-derived hormone) adiponectin in obese patients: adiponectin-knockout mice appear more susceptible to doxorubicin-induced cardiac contractile dysfunction, whilst exogenously-administered adiponectin protects such mice, and enhances cardiac function in wild-type mice treated with doxorubicin (reviewed in [27]).

The American Society of Clinical Oncology’s (ASCO’s) 2012 practice guideline endorses BSA-adjusted dosing of anthracyclines, and advise against empiric dose capping or using ideal body weight for calculations in obese patients [20]. It should be noted, however, that a paucity of toxicity and efficacy data means that these recommendations are not underpinned by a strong evidence base, but rather by concerns that alternative methods result in under-dosing and worse cancer outcomes [20]. Our observation of associations between anthropometric factors and cardiotoxicity, in a mechanistic study, do not change that assessment, but they do warrant further investigation and underscore the need for additional research into optimal dosing strategies.

### Influence of elevated blood pressure on cardiotoxicity risk

Hypertension was first suggested to increase cardiac susceptibility to anthracyclines in the 1970s [28]. However, there have been conflicting reports [22, 29] and it is only in the past decade that studies using large datasets from the Surveillance, Epidemiology and End Results (SEERS)-Medicare database have established hypertension as an independent risk factor, hazard ratios of 1.8 and 1.5 being reported in treatment of lymphoma [10] and early breast cancer [30] respectively. Despite this, interpretation of the literature remains complicated because few data, if any, fully discriminate the contribution of hypertension *per se* to cardiotoxicity, from the influence of associated comorbidities (including coronary disease, atrial fibrillation, diabetes, renal dysfunction, obesity [10, 29, 31, 32]) and cardiovascular medications (such as ACE inhibitors, mineralocorticoid receptor antagonists and beta-blockers) which may be cardioprotective [33, 34]. Our selected cohort allowed prospective study of the influence of blood pressure, largely unhindered by these considerations. Women with elevated BP (≥140/90mmHg) pre-chemotherapy were substantially more likely (OR 5.36 [1.73–17.61]) to be in the *cAC* group, despite mean blood pressure (147.3/86.1mmHg) being only modestly above normal. Although, confirmed clinic measurements ≥140/90mmHg define hypertension [35], blood pressure is a continuous rather than categorical variable. Increasing values from within the normal range correlate with risk of both vascular mortality [36] and heart failure [37]. It thus seems likely that blood pressure also acts as continuous variable in respect of *cAC* risk, albeit that our dataset was too small to be able to investigate this effect. Our results, taken with the wider published literature, suggest that close attention be paid to blood pressure measurement before (and after) chemotherapy, with early treatment initiated when hypertension is confirmed–an approach advocated by expert panels of the European Societies of Cardiology and Medical Oncology–albeit that benefits remain to be proven [1, 6].

### Trastuzumab and cardiotoxicity

We sought clinical features which might contribute to risk of anthracycline-induced (Type 1, permanent) cardiotoxicity. Trastuzumab, whilst not the primary focus of attention, can itself cause cardiotoxicity by both potentiating anthracycline-induced (type I) cardiotoxicity and, more commonly, by causing reversible falls in cardiac contractility (type II cardiotoxicity) [3]. In trials of adjuvant Trastuzumab, LVEF was depressed by ≥10 or ≥15% (depending on study) in 3–16%, while cardiotoxicity led to treatment discontinuation in 4.3–15.6% [1, 6]. Non-trial data suggest an incidence of heart failure/cardiomyopathy as high as 20% 3–5 years after adjuvant Trastuzumab[31].

In our study, all 31 patients commencing Trastuzumab completed the full 12-month course, with one (3.2%) identified as experiencing a transient fall in LVEF *(type II cardiotoxicity)* on routine scanning by clinical teams. None exhibited overt anthracycline cardiotoxicity at follow-up CMR. Trastuzumab was identified as a risk factor for the *subclinical cAC group* on multivariate analysis, albeit that the magnitude of associated risk (OR 3.35 [1.18–9.51]) also reflected the longer interval to follow-up. This relatively muted effect of Trastuzumab is likely a consequence of the low prevalence of comorbidities which are believed to underlie the frequent cardiotoxicity observed in registries [31]. The interval between completing anthracycline and commencing Trastuzumab therapy (median of 3.7 months in our study) is also believed to be important, cardiotoxicity rates being highest when these drugs are co-administered, rather than sequentially administered as was the case for our cohort [6].

### Age, radiotherapy and cardiotoxicity risk

The reported association between older age and cAC [10, 32] was absent in our cohort, likely due to its relative youth (only 0.13% were ≥65 years old), and possibly the low prevalence of age-related comorbidity. The use of radiotherapy was also not associated with a fall in LVEF, consistent with other contemporary reports and probably reflecting the low cardiac-doses received with modern breast cancer treatment [30, 31, 38].

### Risk prediction modelling

Risk prediction models for *cAC* might help guide individual management [3, 8]. Although models have been proposed for *cAC* in metastatic breast cancer and for Trastuzumab cardiotoxicity [21, 31, 32], these are not in widespread clinical use. Meanwhile, none yet exist for *cAC* prediction in patients with early breast cancer. In our study, multivariate analysis revealed the number of anthracycline cycles, body surface area, BP ≥140/90mmHg and Trastuzumab treatment to be the independent discriminators of being in the *cAC* group. The model based on these variables had an area under the ROC curve of 0.78. Predictive tests with an area of >0.7 are generally considered to offer fair discriminatory ability. However, even within our highly selected cohort, the model had a limited positive predictive value (PPV) for cardiotoxicity (62.5% at a 5% false positive rate). To inform therapeutic strategy, models would need to perform significantly better. Despite this, a basic predictive model such as ours might have utility in stratifying patients post-chemotherapy where ongoing cardiac surveillance is considered [8]. While such surveillance is not routine practice (or validated for cost-efficacy), such follow-up has been suggested by both European and American experts [1, 3].

### Subclinical cardiotoxicity and its potential clinical relevance

Given that the contractile reserves of the heart are high, significant cardiotoxicity can be present before LVEF falls by as much as 10% to below normal[5]; furthermore, once such dysfunction has developed it may not be fully reversible [14]. This has led to a search for techniques to allow detection of cardiotoxicity at an earlier stage.

We sought subclinical cardiotoxicity within a selected cohort as a means to identify risk factors and explore disease mechanism. Overt cardiotoxicity is an unsuitable endpoint for our purposes, given scarcity of early dramatic declines, that would have demanded a study of massive scale, and inclusion of more diverse population. The definition of ‘subclinical cardiotoxicity’ has no consensus. However, this does not impact on the validity of our findings as the threshold set will depend on the power to reliably detect cardiotoxicity-related-changes, and thus vary by the phenotype measured (be that LVEF, tissue strain, or biomarkers) and the modality used to do so. We used a fall in LVEF≥5% on CMR to define our subclinical cardiotoxicity group. Although this cut-off is below the threshold recommended for use in clinical practice and in oncology trials (i.e. falls in LVEF of at least 10% to below normal) [1, 14], that threshold reflects the need to diagnose cardiotoxicity in individuals of sufficient severity that interruption of anticancer treatment is warranted–an issue entirely different from that which we addressed. Further, a 10% fall in LVEF is at the limit of what can be reliably detected using 2D echocardiography, the most widely available imaging modality [14]. Our use of CMR (the ‘reference standard’ for LVEF evaluation[14]), allowed us to reliably detect smaller changes in LVEF, given its superior inter-study reproducibility [11, 17]. Meanwhile, others have suggested a fall in LVEF≥5% be used to define anthracycline cardiotoxicity[39]–that report being based on multi-gated acquisition (MUGA), which has high inter-study reproducibility, comparable to CMR (and superior to 2D echocardiography) [14, 15, 39]).

By showing associations with several established cardiotoxicity risk factors, the results from our study validate a fall in LVEF ≥5% as a definition of subclinical cardiotoxicity. Furthermore, we have also validated a study-model which can be applied to larger and more disparate cohorts. Future studies of similar design might be used to study the influence of other factors on cAC risk (such as obesity, physical activity or blood pressure as a continuous variable), or to investigate the efficacy of potentially cardioprotective drugs.

In the short and medium term, changes in left ventricular ejection fraction of the magnitude we observed are not of clinical importance and the benefits of anthracycline/Trastuzumab therapy undoubtedly outweigh the risks in patients such as ours. However, whether and to what extent therapy might impact on cardiac health in the true long-term (potentially several decades hence) is uncertain. In the most comprehensive study in adults to date, Cardinale and colleagues reported a 9% incidence of anthracycline cardiotoxicity (LVEF fall >10% to below 50%) in breast cancer and lymphoma patients undergoing serial echocardiography after a median 5.2 years [38]. Half the patients crossing their diagnostic threshold for cardiotoxicity did so within 3.5 months of completing chemotherapy, and 98% within a year. Nevertheless, a small number of cases of cardiotoxicity developed between 5 and 10 years post chemotherapy, consistent with the progressive nature of anthracycline cardiotoxicity (evidence for which we also identified—above)[38]. This effect, at least in part, explains the increasing prevalence of cardiac dysfunction and heart failure in survivors of childhood cancer over subsequent decades [2, 38, 40, 41]. The mechanisms underlying this phenomenon remain to be fully elucidated given the multiple toxic effects of anthracyclines, but mitochondrial dysfunction (DNA damage and impaired bioenergetics [42]) and possibly cellular senescence (including shortening of telomere length and loss of cardiac progenitor cells [43]) may play a role. Meanwhile, declines in cardiac function after chemotherapy may also occur as a result of “multiple hits”: exposure to subsequent insults, such as coronary ischaemia, hypertension and impaired glucose homeostasis, the prevalence of which is high in cancer survivors [5, 41, 44]. Given these observations, and that falls in resting LVEF imply significant cardiac injury [5], it is quite plausible that those who have experienced falls in LVEF ≥5% following anthracyclines will have an increased lifetime risk of multifactorial heart failure. Even were individual risk low, the number of long-term survivors from breast cancer is large and expanding [8, 38]. Consequently, any small increase in population risk may result in a significant number of additional heart failure cases.

We do not advocate using an acute fall in LVEF≥5% clinically to guide chemotherapy, given that doing so would risk withholding potentially lifesaving chemotherapy.

### Late gadolinium enhancement

Using LVEF alone at any-cut off to diagnose cardiotoxicity in individuals has well-documented limitations [5]. The ability to detect interstitial myocardial fibrosis (a hallmark of advanced anthracycline cardiomyopathy [45]) with CMR might offer additional prognostic value [46]. We observed no late gadolinium enhancement in the 120 cases imaged, confirming this technique is not useful for detection of early *cAC* [46]. However, it does not imply an absence of fibrosis: enhancement is only observed when *focal* areas of fibrosis are present. Newer CMR T1-mapping techniques (not available in the study period) may more accurately detect diffuse fibrosis [46].

### Limitations

Our study does have limitations. Firstly, the cohort is of modest size. This is partially mitigated by prospective assessment using CMR (our study of 165 equates to >1000 subjects had 2-dimensional echocardiography been used [11, 17]), and discriminative power provided by the highly selected (homogenous) cohort. Even so, it is not possible to fit complex models and interactions of covariates using the data from studies of this size. Secondly, we use a subclinical endpoint, and assume that studying risk factors for subclinical *cAC* will give insights into the pathogenesis of more overt cardiotoxicity. Our ability to detect associations with established risk factors support this assumption. Thirdly, being selected by sex, race and for low incidence of cardiovascular co-morbidities (including known coronary artery disease or formally-diagnosed hypertension), the cohort does not accurately reflect the larger population of cancer patients, in whom the absolute risk of *cAC* is likely to be greater, and the relative contribution of the risk factors we identified remains to be confirmed. Fourthly, while we used the standard definition for elevated blood pressure, our measurements were insufficient to formally diagnose hypertension (for which repeated clinic or ambulatory measurements are required)[35]. Indeed, in seeking a homogeneous cohort with low prevalence of cardiovascular comorbidities (above) we had actively sought to exclude such patients. Fifthly, some datasets were not entirely complete: percentage body fat was only available in 118; blood pressure was unavailable for 1 subject and measured at a single time-point in 14%; and radiotherapy details were unavailable in 2%. In addition, the interval between chemotherapy and follow-up varied (largely due to difficulties in patients prioritising return). However, we view none of these factors as systemic confounders to the validity of our findings. Finally, our model was developed and tested on the same population and the ROC curve area may therefore be overestimated. Validation on a separate sample is required to confirm the predictive ability.

## Conclusions

In conclusion, by using CMR to measure LVEF, we were able to detect evidence of subclinical cardiotoxicity in 20% of our low risk cohort. Further, we were able to demonstrate associations between falls in LVEF ≥5% and known risk factors for *cAC*—most importantly the characteristic cumulative dose-dependence. This confirms the high discriminative power of our study-model, despite its relatively modest size. Future studies using a similar design might be used to further explore the cardiotoxicity of anthracyclines: for example, the influence of obesity, of old-age (independent to age-related comorbidity), of physical activity, or the efficacy of potentially cardioprotective drugs. On multivariate analysis, the number of anthracycline cycles, BSA, Trastuzumab use and BP≥140/90mmHg were independent predictors of subclinical *cAC*. The model based on these factors had an area under the ROC curve of 0.78. While this equates to reasonable discriminative value, the predictive value is insufficient to reliably identify individuals with cardiotoxicity even within our selected cohort. Nevertheless, our results do offer potential mechanistic insights. Given that *cAC* was associated with only modestly elevated BP, we recommend recognition of hypertension and initiation of therapy before commencing anthracycline chemotherapy as prudent. Finally, the association between increasing BSA and subclinical *cAC* risk suggests that BSA-indexing of anthracycline dose may be causal—raising the question as to whether this approach is optimal for all, and pointing to the need for additional research in this area.

## Supporting Information

S1 Table Treatment data. (DOCX)

S2 Table LV volume and mass data. (DOCX)
