# Supplementary material for: Body Surface Area and Baseline Blood Pressure Predict Subclinical Anthracycline Cardiotoxicity in Women Treated for Early Breast Cancer
Source: PLoS One. 2016 Dec 2;11(12):e0165262. doi: 10.1371/journal.pone.0165262 (PMC5135038; doi:10.1371/journal.pone.0165262)
Supplement: S1 Table — (DOCX) [file pone.0165262.s001.docx]

| **Table S1: Treatment data** | |
| --- | --- |
| **Treatment** | **% , or**  **Median [IQR]** |
| **Anthracyclines** | |
| Epirubicin | 98.8% |
| Doxorubicin | 1.2% |
| Actual cumulative dose of epirubicin (mg)^*^ | 698 [564-780] |
| Cumulative dose of epirubicin / BSA (mg/m^2^) | 400 [300-450] |
| Number of anthracycline cycles | 4 [4-6] |
| **Chemotherapy Regimens** | |
| Epirubicin + Flurouracil + Cyclophosphamide + Methotrexate | 14.6% |
| Epirubicin + Flurouracil + Cyclophosphamide + Docetaxel | 23.0% |
| Epirubicin + Cyclophosphamide + Docetaxel | 4.3% |
| Epirubicin + Capecitabine | 2.4% |
| Epirubicin + Cyclophosphamide + Pacitaxel + Gemcitabine | 2.4% |
| Epirubicin + Cyclophosphamide + Pacitaxel | 1.8% |
| Doxorubicin + Cyclophosphamide + Docetaxel | 0.6% |
| Doxorubicin + Cyclophosphamide + Pacitaxel | 0.6% |
| **Other Therapy** | |
| Radiotherapy | 86.3% |
| Left chest wall radiotherapy | 42.9% |
| Trastuzumab (12 month course) | 18.8% |
| Interval anthracycline end to trastuzumab start (months) | 3.7 [1.3-5.1] |
| Aromatase inhibitor | 13.3% |
| Tamoxifen | 52.1% |
| ACE inhibitor, ARB or beta-blocker (prechemotherapy) | 0% |
| ACE inhibitor, ARB or beta-blocker (at follow-up) | 0% |
| Number of anthracycline cycles and cumulative epirubicin dose correlated, r=0.90 (p<0.001) Two subjects received doxorubicin a cumulative dose of 240mg/m^2^ (over 4 cycles) rather than epirubicin. Cumulative Epirubicin dose data incomplete for one patient who emigrated (having received 270mg/m^2^). Details of radiotherapy not available in 4 patients. One patient was treated with an ACE inhibitor during Trastuzumab therapy following an asymptomatic decline in LVEF on echocardiography (Trastuzumab course completed and ACE inhibitor discontinued 12 months prior to follow-up). | |
