# Supplementary material for: Body Surface Area and Baseline Blood Pressure Predict Subclinical Anthracycline Cardiotoxicity in Women Treated for Early Breast Cancer
Source: PLoS One. 2016 Dec 2;11(12):e0165262. doi: 10.1371/journal.pone.0165262 (PMC5135038; doi:10.1371/journal.pone.0165262)
Supplement: S2 Table — (DOCX) [file pone.0165262.s002.docx]

**Supplementary Table S2: Left ventricular volume and mass**

| Variable | Group | N | Baseline SD | | Follow-up SD | | Mean  Change 95% CI p-value^†^ | |
| --- | --- | --- | --- | --- | --- | --- | --- | --- |
| EDV/BSA (ml/m^2^) | combined | 165 | 64.39 | 9.06 | 63.90 | 8.77 | -0.49 | 0.14 |
|  | cAC group | 34 | 63.69 | 7.42 | 64.30 | 8.01 | 0.61 | 0.53 |
|  | Minimally/unaffected group | 131 | 64.57 | 9.46 | 63.80 | 8.98 | -0.77 | 0.02 |
|  |  | p-value^*^ | 0.61 |  | 0.77 |  | 0.09 |  |
| Mass / BSA (g/m^2^) | combined | 165 | 63.07 | 7.09 | 62.79 | 7.10 | -0.28 | 0.36 |
|  | cAC group | 34 | 63.59 | 7.00 | 64.89 | 6.93 | 1.30 | 0.09 |
|  | Minimally/unaffected group | 131 | 62.93 | 7.13 | 62.24 | 7.07 | -0.69 | 0.03 |
|  |  | p-value^*^ | 0.63 |  | 0.052 |  | 0.007 |  |
| Data presented indexed to body surface area (BSA). EDV: end diastolic volume. Change between baseline and follow-up was tested with paired t-tests (p-value^†^). The difference between groups was assessed using unpaired t-tests (p-value^*^). Normally left ventricular EDV and mass both fall overtime as a result of age related remodel (12,16). This normal pattern was observed in the minimally affected group but not the cAC group. The small rise in mass seen in the cAC group was not significant from baseline, however the response was significantly different when compared to the fall seen in the minimally /unaffected group. | | | | | | | | |
